# H^+^-pyrophosphatases enhance low nitrogen stress tolerance in transgenic *Arabidopsis* and wheat by interacting with a receptor-like protein kinase

**DOI:** 10.3389/fpls.2023.1096091

**Published:** 2023-01-27

**Authors:** Huijuan Zhang, Ming Chen, Chengjie Xu, Rongbang Liu, Wensi Tang, Kai Chen, Yongbin Zhou, Zhaoshi Xu, Jun Chen, Youzhi Ma, Weiguo Chen, Daizhen Sun, Hua Fan

**Affiliations:** ^1^ College of Agriculture, Shanxi Agricultural University, Shanxi, China; ^2^ Institute of Crop Sciences, Chinese Academy of Agricultural Sciences (CAAS)/National Key Facility for Crop Gene Resources and Genetic Improvement, Key Laboratory of Biology and Genetic Improvement of Triticeae Crops, Ministry of Agriculture, Beijing, China

**Keywords:** low nitrogen stress, receptor-like protein kinase, arabidopsis, transgenic wheat, H^+^-pyrophosphatase

## Abstract

**Introduction:**

Nitrogen is a major abiotic stress that affects plant productivity. Previous studies have shown that plant H+-pyrophosphatases (H+-PPases) enhance plant resistance to low nitrogen stress. However, the molecular mechanism underlying H+-PPase-mediated regulation of plant responses to low nitrogen stress is still unknown. In this study, we aimed to investigate the regulatory mechanism of AtAVP1 in response to low nitrogen stress.

**Methods and Results:**

AtAVP1 in Arabidopsis thaliana and EdVP1 in Elymus dahuricus belong to the H+-PPase gene family. In this study, we found that AtAVP1 overexpression was more tolerant to low nitrogen stress than was wild type (WT), whereas the avp1-1 mutant was less tolerant to low nitrogen stress than WT. Plant height, root length, aboveground fresh and dry weights, and underground fresh and dry weights of EdVP1 overexpression wheat were considerably higher than those of SHI366 under low nitrogen treatment during the seedling stage. Two consecutive years of low nitrogen tolerance experiments in the field showed that grain yield and number of grains per spike of EdVP1 overexpression wheat were increased compared to those in SHI366, which indicated that EdVP1 conferred low nitrogen stress tolerance in the field. Furthermore, we screened interaction proteins in Arabidopsis; subcellular localization analysis demonstrated that AtAVP1 and Arabidopsis thaliana receptor-like protein kinase (AtRLK) were located on the plasma membrane. Yeast two-hybrid and luciferase complementary imaging assays showed that the AtRLK interacted with AtAVP1. Under low nitrogen stress, the Arabidopsis mutants rlk and avp1-1 had the same phenotypes.

**Discussion:**

These results indicate that AtAVP1 regulates low nitrogen stress responses by interacting with AtRLK, which provides a novel insight into the regulatory pathway related to H+-pyrophosphatase function in plants.

## 1 Introduction

Nitrogen (N) is an essential macronutrient for plants to achieve optimal growth and development; therefore, low nitrogen is often a major limiting factor for crop yield (Jones et al., 2013). In actual crop production systems, excessive nitrogen fertilizer is used to maintain high yields. The extensive application of nitrogen fertilizer not only increases the cost of crop production but also causes serious environmental problems (Guo et al., 2010). In fact, only 30–50% of applied nitrogen is absorbed by the crop plants (Zhu et al., 2018). Improving crop nitrogen use efficiency (NUE) can increase crop productivity with less nitrogen fertilizer input (Han et al., 2015). H^+^-pyrophosphatases (H^+^-PPases) can respond to various abiotic stresses, including low nitrogen stress. For example, overexpression of *Arabidopsis thaliana AVP1* (*AtAVP1*) has been reported to improve the NUE in *Romaine Lettuce* (Paez-Valencia et al., 2013).

H^+^-PPases are hydrolases that use pyrophosphate (PPi) as a substrate. In plants, there are type I and type II H^+^-PPases. At present, the composition and structure of H^+^-PPase is mainly a vacuolar membrane topological model, consisting of a single oligopeptide chain (Maeshima, 2000). Type II H^+^-PPase is mainly distributed in the Golgi apparatus, and the Type II H^+^-PPase accounts for 0.2% of the total type I H^+^-PPase. The type I H^+^-PPase was mainly located in the vacuolar membranes, but in recent years type I H^+^-PPases was also located on the plasma membrane (PM) in *Ricinus communis, cauliflower, Oryza sativa* and *Arabidopsis thaliana* (Serrano et al., 2007; Paez-Valencia et al., 2011). Preliminary studies show that H^+^-PPases are generally expressed in young, growing tissues, and are highly expressed in *Arabidopsis thaliana* under drought and salt stress (Shiratake et al., 1997; Paez-Valencia et al., 2011). The lack of the a2 and a3 subunits of another tonoplast proton pump V-ATPase resulted in an increase in its nitrate reductase activity under 24 h light (Krebs et al., 2010). H^+^-PPase acts as a proton pump along with vacuolar V-ATPase to maintain acidic pH within the vacuolar lumen (Yoichi et al., 2003). Further the functional and physical characteristics are different (Maeshima, 2000).The activity of tonoplast proton pump V-ATPase creates the proton gradient and the membrane potential that is used to transport compounds against their concentration or electrochemical gradients (Schumacher, 2007). And Li et al. showed that the absorption of ${\text{NO}}_{3}^{-}$ concentrations regulated by *TaVP* (H^+^-pyrophosphatase) were caused by increased root absorption area instead of alteration of Pi and ${\text{NO}}_{3}^{-}$ acquisition kinetics (Li et al., 2014).

The overexpression of *AVP1* was also reported to improve salt tolerance and drought tolerance of transgenic *Arabidopsis* (Gaxiola et al., 2001). Additionally, the drought and salt tolerance of transgenic plants was significantly improved when H^+^-PPases from different plants (including *Arabidopsis*), and other type I H^+^-PPases were overexpressed in alfalfa (Bao et al., 2009; Su et al., 2019), barley (Schilling et al., 2014), cotton (Pasapula et al., 2011), and tobacco (Gao et al., 2006). H^+^-PPases also respond to low phosphorus stress in plants. When H^+^-PPase genes were expressed in tomato, rice (Yang et al., 2007), tobacco (Li et al., 2014) and alfalfa (Su et al., 2019), the plants grew better in low phosphorus environment than did wild type (WT). In addition, *AVP1* can improve low nitrogen tolerance in plants. *TaAVP* overexpressed in tobacco increased the nitrogen content in the transgenic plants to an extent more than that observed in WT tobacco (Li et al., 2014). In our previous study, we found that the *Elymus dahuricus* H^+^-PPase gene (*EdVP1*) was induced by low potassium stress, and overexpression of *EdVP1* in wheat considerably increased the grain yield and potassium uptake in transgenic wheat under low potassium stress (Zhou et al., 2020). These findings show that H^+^-PPases genes play an important role in plant response to various abiotic stresses. However, our understanding of the regulatory mechanisms related to H^+^-pyrophosphatases is still very limited.

In this study, we aimed to investigate the regulatory mechanism of *AtAVP1* in response to low nitrogen stress. We found that the H^+^-pyrophosphatase (*AVP1*) gene could significantly improve plant resistance to low nitrogen stress in *Arabidopsis*, and overexpression of the *Elymus dahuricus* homolog of *AtAVP1* in wheat could also significantly improve the resistance of transgenic wheat to low nitrogen stress in greenhouse and field conditions. Furthermore, we found that the receptor-like kinases in *Arabidopsis* (AtRLK) can interact with AtAVP1, and the *Arabidopsis* mutant *rlk* had similar sensitivity to low nitrogen stress as the *Arabidopsis* mutant *avp1-1*. Our study provides useful insights into genes that may contribute towards strengthening plant tolerance against abiotic stress, and would help to improve crop plant production and associated costs.

## 2 Material and methods

### 2.1 *Arabidopsis* plants and growth conditions

For phenotypic analysis of transgenic, mutant and WT *Arabidopsis thaliana* plants under stress, we used the *Arabidopsis* ecotype Columbia-0 as WT and the *avp1-1* (SALK_019677) and *rlk* (SALK_123639C) mutants, which were obtained from the *Arabidopsis* Biological Resource Center (Ohio State University, Columbus, OH, USA). Mutants were screened *via* the triple primer method, using the T-DNA Primer Design tool (http://signal.salk.edu/tdnaprimers.2.html) (mutants represent T-DNA insertion). Quantitative reverse transcription-PCR (qRT-PCR) was used to detect the expression of *AtAVP1* and *AtRLK* (AT5G35370) in WT and transgenic plants. Transgenic *AtAVP1 Arabidopsis* (OE-*AtAVP1*) plants were obtained using the floral dip method (Clough and Bent, 1998). The coding sequence (CDS) of *AtAVP1* (AT1G15690) was retrieved from the TAIR website (https://www.Arabidopsis.org/). The CDS of *AtAVP1* was inserted into the binary vector pCAMBIA1302 driven by a CaMV35S promoter, following which the circular plasmids were digested with *NcolⅠ*, and the recombinant binary vector pCAMBIA1302-AtAVP1 was cloned into the *Agrobacterium tumefaciens* strain GV1301, which as then used to transform plants using the floral dip method (transgenes represent overexpression of the 35S promoter). Homozygous T_3_ lines were identified *via* PCR detection. The primers used in this study are shown in **Supplementary Table 1**.

The seedlings of *Arabidopsis* (WT, OE-*AtAVP1*, *avp1-1*, and *rlk*) were sterilized with 10% sodium hypochlorite for 10 min and then washed with sterile water five times for 1 min each wash. Sterilized seeds were sown on MS_0_ medium (Millipore Sigma, St. Louis, MO, USA) and placed in a growth chamber (16 h light/8 h dark, 22 °C, light intensity around 100 μ·m^-2^·s^-2^) for 3 d before being transferred to 6 mM low ${\text{NO}}_{3}^{-}$ (control), 1 mM low ${\text{NO}}_{3}^{-}$ , or 0.2 mM ${\text{NO}}_{3}^{-}$ (low nitrogen) medium (Ma et al., 2014), following which they were grown at a 30° angle. Low nitrogen media were prepared using Murashige & Skoog (MS) modified basal salt mixture (no nitrogen, phosphate, or potassium) reagent preparation M407 purchased from Phyto Technology Laboratories ™. For control medium, 6mM ${\text{NO}}_{3}^{-}$ were applied to the medium without nitrogen, and 1 and 0.2 mM ${\text{NO}}_{3}^{-}$ were applied externally to the treatment medium. Specific ingredients are shown in **Supplementary Table 2**. The seedlings were photographed when phenotypic differences were observed. Win RHIZO Pro 2012 root scanning software (Regent Instrument Inc., Ottawa, ON, Canada), EPSON flatbed scanner, and Expression 10000XL scanners were used to scan and measure root length, total root area, and lateral root number. Each treatment was conducted in triplicate, with each line tested in at least six plants.

The *Arabidopsis* ecotype Columbia-0 WT was used for gene expression analysis. First, the *Arabidopsis* seeds were sterilized and cultured as described above; then, the seedlings with uniform growth were transferred to a medium containing nitrogen, indole acetic acid (IAA) and salt (NaCl). For IAA and NaCl treatments, MS_0_ medium containing the following (all from Millipore Sigma, St. Louis, MO, USA) were used. The final concentrations of IAA were 0 (MS_0_), 1 μM, and 10 μM (Guo et al., 2002; Zheng et al., 2017). For the NaCl treatment, the final concentrations were 50 mM and 100 mM. Roots were harvested after 7 d of treatment to detect changes in gene expression in response to different stresses. These experiments were conducted in triplicate for each treatment.

### 2.2 Sequence alignment and phylogenetic tree construction of *AtAVP1*

The *AtAVP1* and *EdVP1* exon-intron substructures were mapped using the server (GSDS) program (http://gsds.cbi.pku.edu.cn/). Genome and protein sequences were obtained from the TAIR website (https://www.Arabidopsis.org/) through a keyword search for *AtAVP1*. The NCBI BLAST site (https://blast.ncbi.nlm.nih.gov/Blast.cgi) was used to find and download gramineous protein sequences highly homologous to AtAVP1. The AtAVP1 phylogenic tree was constructed using the neighbor-joining (NJ) method *via* the MEGAX software (https://www.megasoftware.net/dload_win_gui) as previously described (Kumar et al., 2016). Protein sequence alignment was performed using DNAMAN.

### 2.3 Analysis of low nitrogen tolerance in greenhouse-grown *EdVP1* transgenic wheat

The transgenic *EdVP1* wheat lines OE-8, OE-28, and OE-32 were previously constructed in the laboratory (Zhou et al., 2020). These lines were self-preserved in our laboratory. Genomic DNA was isolated from OE-EdVP1 lines and SHI366 wheat plants using the CTAB method (Kim et al., 2005) and then analyzed *via* PCR. Homozygous transgenic lines were screened using PCR. qRT-PCR was used to detect the expression of *EdVP1* in WT, OE-8, OE-28, and OE-32 lines. The primers used are shown in **Supplementary Table 1**.

In the hydroponic low nitrogen tolerance experiments, transgenic wheat seeds with consistent germination were selected and transplanted into the pots. The seedlings were cultivated in a greenhouse (60% relative humidity) with 14 h light at 25 °C and 10 h darkness at 20 °C. After 3 d, the seedlings were cultured hydroponically in nutrient solutions with a control medium concentration of 2 mM ${\text{NO}}_{3}^{-}$ and a low nitrogen medium concentration of 0.2 mM ${\text{NO}}_{3}^{-}$ . The nutrient solutions used for the hydroponic phase were used as described previously (Qu et al., 2015).The nutrient solution was changed daily. Five plants from each treatment were randomly selected for determining various indices after one week, including plant height, root length, aboveground fresh and dry weight, and underground fresh and dry weight. Each experiment was repeated thrice.

### 2.4 Analysis of low nitrogen tolerance in transgenic wheat under field conditions

The transgenic lines OE-28 and OE-32 showed better performance in the laboratory and, therefore, were selected for low nitrogen field experiments. All experiments were conducted at the Shun Yi experimental station of the Institute of Crop Sciences of the Chinese Academy of Agricultural Sciences in Beijing. Two consecutive field experiments were conducted in 2020-2021 using the transgenic T_4_ generation and in 2021-2022 using the transgenic T_5_ generation. Both experiments comprised two treatments, each with three replicates. Both treatments included 7.5 g·m^-2^ P in the form of superphosphate. The normal nitrogen treatment had 15.6 g N·m^-2^ in the form of urea with 8.9 g N·m^-2^ applied prior to sowing and 6.7 g N·m^-2^ applied at the stem elongation stage. The low nitrogen treatment had no nitrogen application. Plot size was 6 m^2^ (4 m × 1.5 m), which included six rows spaced 25 cm apart; the sowing density was 300 germinating seeds per m^2^. At maturity, twenty spikes in each plot were randomly collected to measure grain number per spike, and the 1000-grain weight. Spike numbers in two 1-m rows of each plot were recorded, and the spike number per square meter was calculated. The yield was calculated using the actual grain weight of each plot by converting the plot area.

### 2.5 Yeast two-hybrid system for screening AtAVP1 protein interactions

The yeast two-hybrid system was used to screen the *Arabidopsis* cDNA library (Stagljar et al., 1998). The yeast library screening for this experiment was carried out by the Oyman Biotech company (Oebiotech, Shanghai, China). The CDS sequence of *AtAVP1* was amplified from the cDNA and ligated into the pBT3-STE vector to construct the bait vector. After validating the autoactivation activity of the vector, the cDNA library was screened; the *Arabidopsis* cDNA library plasmid was then cloned into NMY51 yeast cells. Blue/white screening for colonies was performed on an SD/-Leu-Trp-His-Ade medium containing X-α-gal. The sequences of blue yeast colonies were subjected to NCBI BLAST. Library screening and reagent preparation were performed according to the system method (http://www.dualsystems.com/).

The interaction between AtAVP1 and AtRLK was verified with the ubiquitin isolation system. The coding regions of *AtAVP1* were inserted into the bait vector pBT3-STE, and the full-length CDS of *AtRLK* was inserted into the capture vector pPR3N. The primers used are shown in **Supplementary Table 1**. The bait vector pBT3-STE : AtAVP1 and the capture vector pPR3N:AtRLK were co-transformed into NMY51 yeast cells. The yeast cells were spread onto SD/-Leu-Trp and SD/-Leu-Trp-His-Ade-deficient medium plates and cultured at 30 °C for 3-4 d. Unseeded plates were used as the corresponding negative controls.

### 2.6 Luciferase complementary imaging assay

The CDSs of *AtAVP1* and *AtRLK* were inserted into the pCAMBIA1300-nLUC and pCAMBIA1300-cLUC vectors to obtain nLUC-AtAVP1 and cLUC-AtRLK recombinant plasmids, respectively. The primers used are shown in **Supplementary Table 1**. These recombinant plasmids were cloned into *Agrobacterium tumefaciens* (GV3101) and cultured to a final concentration of OD_600_ = 0.2; the nLUC-AtAVP1/cLUC-AtRLK, nLUC/cLUC, nLUC-AtAVP1/cLUC, and nLUC/cLUC-AtRLK combinations of *A. tumefaciens* were then infiltrated into tobacco leaves. After 48 h, the leaves were analyzed with Night SHADE LB 985 (Berthold Technologies, Germany). These experiments were conducted at least three times.

### 2.7 Subcellular localization of AtAVP1 and AtRLK

The coding regions of *AtAVP1* and *AtRLK* (with the termination codon removed) were inserted into the p16318hGFP vector for GFP-protein fusion expression. The resultant 35S:AtAVP1-GFP and 35S:AtRLK-GFP vectors driven by a CaMV35S promotor were constructed and used for subcellular localization studies. The primers used are shown in **Supplementary Table 1**. The 35S:AtAVP1-GFP recombinant plasmid and mCherry vectors (the signals of mCherry proteins were located in the membrane) harboring membrane-localized proteins were co-transformed into *Arabidopsis* protoplasts as previously described (Abel and Theologis, 1994). respectively, and cultured at 25 °C under dark conditions for more than 18 h. The expression of the vectors was observed with a Zeiss LSM980 confocal laser scanning microscope.

### 2.8 RNA extraction and qRT-PCR

Total RNA was extracted according to the Plant RNA Extraction Kit manual (Zoman Biotechnology Co, Beijing, China). This RNA was used as a template for synthesizing cDNA using the TransScript One-Step gDNA Removal and cDNA Synthesis SuperMix Kit (TransGene, Beijing, China). A real-time fluorescence quantitative detection kit (TransGene, Beijing, China) and the ABI 7500 real-time quantitative PCR instrument (Thermo Fisher, Waltham, MA, USA) was used to detect the fluorescence signal. The primers used are shown in **Supplementary Table 1**. The *Arabidopsis* reference gene was AT3G18780.The 2^-△Ct^ method was used to calculate the relative expression of genes according to the Ct value of each sample at a specific fluorescence threshold.

### 2.9 Data analysis

All data were analyzed *via* a one-way ANOVA using SPSS19, and the significance of differences between multiple groups of samples was analyzed using Duncan’s multiple range test. Significance level was *p<* 0.05. All results are shown as mean ± standard deviation (SD).

## 3 Results

### 3.1 Characteristics of transgenic *AtAVP1* and mutant *avp1* plants under low nitrogen stress

**Figure 1:**
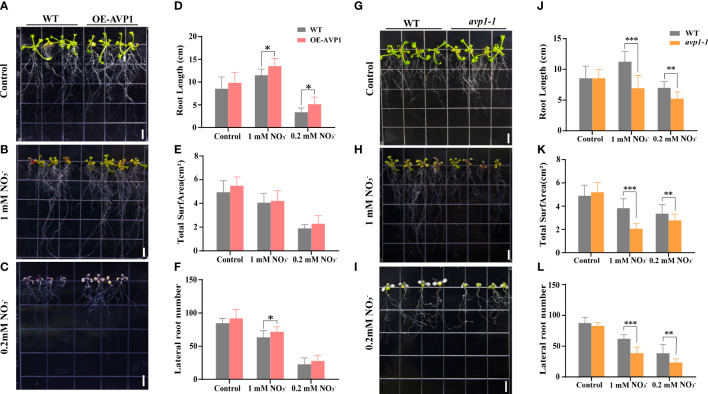
Functions of *AtAVP1* in *Arabidopsis* under normal conditions and different levels of nitrogen stress Phenotypic analysis of the transgenic *AtAVP1 Arabidopsis* line OE-AVP1 grown under **(A)** 6 mM ${\text{NO}}_{3}^{-}$ (control), **(B)** 1 mM ${\text{NO}}_{3}^{-}$ , and **(C)** 0.2 mM ${\text{NO}}_{3}^{-}$ (low nitrogen) conditions. Images were taken after 5 d of growth on different media. DIAN Win RHIZO Pro 2012 root scanning software and EPSON Flatbed and Expression 10000XL scanners were used to scan and measure the roots. **(D)** Root length, **(E)** total root surface, and **(F)** lateral root number of OE-AVP1 lines grown under different nitrogen treatment conditions. Phenotypic analysis of the mutant *avp1-1* under **(G)** 6 mM ${\text{NO}}_{3}^{-}$ (control), **(H)** 1 mM ${\text{NO}}_{3}^{-}$ , and **(I)** 0.2 mM ${\text{NO}}_{3}^{-}$ medium conditions. Images were taken after 7 d of growth; the **(J)** root length, **(K)** total root surface, and **(L)** lateral root number of *avp1-1* lines grown under different nitrogen treatments were measured. All data represent mean ± standard deviation (n ≥ 6). (* *p<* 0.05, ** *p*< 0.01, *** *p*< 0.001, Duncan’s multiple range test). Scale bars = 0.5 cm.

To identify the function of *AtAVP1* under low nitrogen stress, we constructed homozygous transgenic *AtAVP1* (OE-AtAVP1) and mutant *avp1-1 Arabidopsis* lines (**Supplementary Figure 1A, B**). The homozygous mutant was screened by PCR according to the insertion location of T-DNA (**Supplementary Figure 1E**), gene expression was measured in T_3_ generation transgenic plants using qRT-PCR (**Supplementary Figure 1F**). Results showed that the expression of *AtAVP1* was increased in the OE-AVP1 line under 6 mM ${\text{NO}}_{3}^{-}$ , 1 mM ${\text{NO}}_{3}^{-}$ , and 0.2 mM ${\text{NO}}_{3}^{-}$ conditions, and the expression of *AtAVP1* in the mutant *avp1-1* was substantially decreased under 6 mM ${\text{NO}}_{3}^{-}$ , 1 mM ${\text{NO}}_{3}^{-}$ , and 0.2 mM ${\text{NO}}_{3}^{-}$ conditions compared to that in WT plants (**Supplementary Figure 1F**). The growth of OE-AVP1 plants was higher under 1 mM ${\text{NO}}_{3}^{-}$ and 0.2 mM ${\text{NO}}_{3}^{-}$ conditions (**Figures 1B, C**), and the root length of OE-AVP1 plants was significantly higher than that of WT under the same conditions (*p<* 0.05) (**Figure 1D**). Under 0.2 mM ${\text{NO}}_{3}^{-}$ conditions, total root area and lateral root number were higher than those of WT (**Figures 1E, F**). Under 1 mM ${\text{NO}}_{3}^{-}$ conditions, lateral root number were significantly higher than those of WT (*p<* 0.05) (**Figure 1F**). Conversely, the growth of mutant *avp1-1* plant was low under 1 mM ${\text{NO}}_{3}^{-}$ and 0.2 mM ${\text{NO}}_{3}^{-}$ conditions compared with that of WT (**Figures 1H, I**). After treatment with 0.2 mM ${\text{NO}}_{3}^{-}$ and 1 mM ${\text{NO}}_{3}^{-}$ , root length, total root area and lateral root number in the mutant *avp1-1* plants were significantly reduced compared to those in WT (*p<* 0.01) (**Figures 1J–L**); however, no significant differences were observed between WT and OE-AVP1 or between WT and mutant *avp1-1* under normal nitrogen conditions (**Figures 1A, G**). These findings indicate that *AtAVP1* positively regulates tolerance to nitrogen deficiency in *Arabidopsis*.

### 3.2 Transgenic *EdVP1* wheat seedlings have enhanced tolerance to low nitrogen stress under greenhouse conditions

We previously constructed transgenic *EdVP1* wheat plants and found that *EdVP1* from *Elymus dahuricus* improved low potassium tolerance in transgenic wheat grown in the field ( (Zhou et al., 2020). In this study, we analyzed *AtAVP1* and *EdVP1* gene structures (**Supplementary Figure 2A**) and constructed a phylogenetic tree, which showed that EdVP1 had the closest homology with TaAVP1 (**Supplementary Figure 2B**). The protein sequence identity was 97.68% between EdVP1 and TaAVP1 (**Supplementary Figure 2C**). These results indicated that the *AVP1* sequence remained conserved during the evolution of gramineous crops. We further characterized the low nitrogen tolerance of the transgenic *EdVP1* wheat in greenhouse conditions.

**Figure 2:**
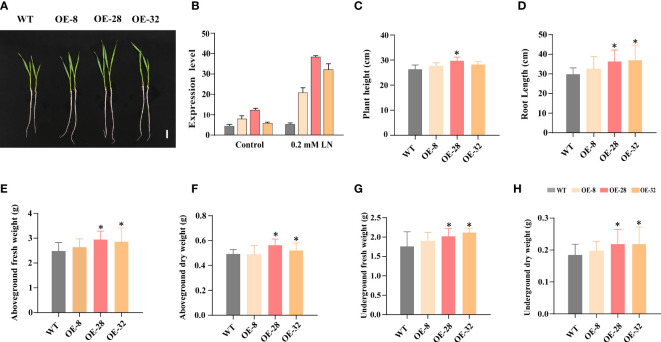
*EdVP1* overexpression in wheat seedlings enhances low nitrogen stress tolerance **(A)** Phenotypes of the WT (SHI366) and transgenic *EdVP1-*overexpressing wheat lines OE-8, OE-28, and OE-32 grown under 0.2 mM low nitrogen (0.2 mM ${\text{NO}}_{3}^{-}$ ) conditions. Scale bars = 5 cm. **(B)** *EdVP1* expression in seedling roots of WT and T_3_ transgenic *EdVP1-*overexpressing wheat lines grown under 0.2 mM ${\text{NO}}_{3}^{-}$ and normal conditions. **(C)** Plant height, **(D)** root length, **(E)** aboveground fresh weight, **(F)** aboveground dry weight, **(G)** underground fresh weight, and **(H)** underground dry weight of WT (SHI366) and the transgenic *EdVP1-*overexpressing wheat lines OE-8, OE-28, OE-32 grown under 0.2 mM low nitrogen condition. Data represent mean ± standard deviation (n = 5), (*p< 0.05, Duncan’s multiple range test).

We screened *EdVP1* T_3_ homozygous transgenic lines using PCR and qRT-PCR, which showed that under 0.2 mM low nitrogen conditions, the transgenic wheat lines OE-8, OE-28, and OE-32 had higher *EdVP1* expression levels than did SHI366 (WT) (**Supplementary Figure 3A**, **Figure 2B**). We analyzed the transgenic wheat grown in the greenhouse and found that under the low nitrogen treatment (0.2 mM ${\text{NO}}_{3}^{-}$ ), the plant height of the line OE-28 was significantly higher than that of WT (*p<* 0.05) (**Figures 2A, C**). The root length, aboveground fresh and dry weight, and underground fresh and dry weight of transgenic wheat lines OE-28 and OE-32 were significantly increased compared with those of WT (*p<* 0.05) (**Figures 2D-H**). The plant height, root length, aboveground fresh and dry weight, and underground fresh and dry weight of the transgenic wheat line OE-8 were increased compared with those of WT (**Figures 2C–H**).

### 3.3 *EdVP1* overexpression enhances the yield of field-grown transgenic wheat under low nitrogen stress

**Figure 3:**
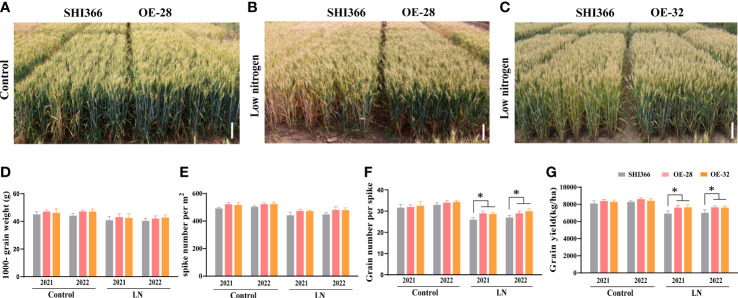
Low nitrogen stress tolerance analysis of *EdVP1* transgenic wheat grown in the field Phenotypes of OE-28 and SHI366 wheat plants grown under **(A)** normal and **(B)** low nitrogen conditions in 2022. **(C)** Phenotypes of OE-32 and SHI366 wheat plants grown under low nitrogen conditions in 2021. Scale bars = 10 cm **(D)** The 1000-grain weight, **(E)** spike number per m^2^, **(F)** grain number per spike, and **(G)** grain yield of the OE-28 and OE-32-overexpressing lines and wild-type SHI366 plants grown under normal and low nitrogen conditions in 2021 and 2022. Data represent the mean of values from three biological replicates ± standard deviation. Asterisks indicate significant differences between the OE-28, OE-32, and SHI366 wheat plants, as assessed using Duncan’s multiple range test (*p< 0.05, Duncan’s multiple range test).

To analyze the role of H^+^-pyrophosphatases in field grown crops, we selected two optimal transgenic lines OE-28 and OE-32 and assessed their low nitrogen tolerance in the field. The low nitrogen tolerance experiments were conducted in Beijing for two consecutive years between 2020-2022. Under low nitrogen stress, OE-28 and OE-32 showed better growth and greener leaves than WT (wheat variety SHI366), while under normal conditions, the transgenic wheat lines OE-28, OE-32 showed similar growth to WT (**Figures 3A–C**). Analysis of the three main yield components revealed that under low nitrogen conditions, OE-28 and OE-32 showed a significant increase in the grain number per spike over WT (*p<* 0.05), while there was no significant difference in spike number and the 1000-grain weight (**Figures 3D–G**). Under low nitrogen stress, the grain yield of OE-28 increased by 10.10% and 9.16%, while that of OE-32 increased by 10.56% and 8.81% compared with that of WT, in 2021 and 2022, respectively (**Figure 3G**, **Supplementary Table 3**). These findings indicate that overexpression of *EdVP1* increased the yield of field-grown wheat under low nitrogen stress, which suggests that the H^+^-pyrophosphatase gene has potential application in increasing grain yield under low nitrogen conditions.

### 3.4 Subcellular localization of AtAVP1 and AtRLK

We screened the proteins interacting with AtAVP1 using the yeast two-hybrid system; the results showed that yeast cells expressing the receptor-like protein kinase AtRLK and AtAVP1 interacted with each other in a heterologous system (**Supplementary Table 4**). Additionally, the expression profile analysis indicated that *AtRLK* was induced by low nitrogen stress in *Arabidopsis* (**Supplementary Figure 1I**).

**Figure 4:**
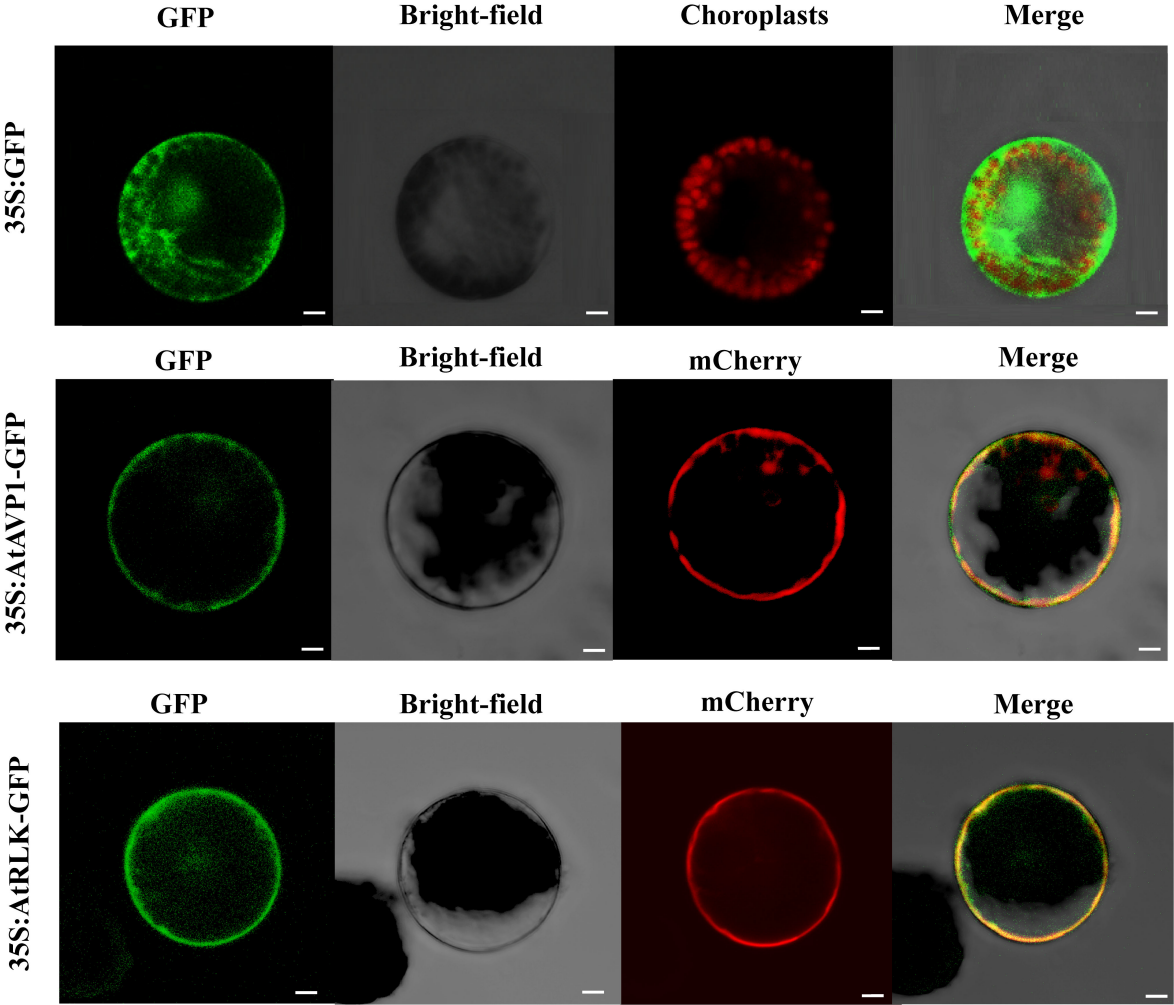
Subcellular AtAVP1 and AtRLK localization in *Arabidopsis* protoplasts The 35S:AtAVP1-GFP, 35S:AtRLK-GFP, and 35S:GFP control vectors were transiently expressed in *Arabidopsis* protoplasts. Compared to that of the 35S:GFP construct, the expression of 35S:AtAVP1-GFP and 35S:AtRLK-GFP was localized to the plasma membrane of the protoplasts. Fluorescence was observed using a Zeiss LSM980 confocal laser scanning microscope 16 h after transformation. Membrane marker protein (mCherry) was co-expressed with 35S:AtAVP1-GFP. Scale bars = 5 μm.

We found that both AtAVP1 and AtRLK were highly expressed in the roots of *Arabidopsis* (**Supplementary Figure 1D**). To further determine subcellular AtAVP1 and AtRLK protein localization, the fusion constructs 35S:AtAVP1-GFP and 35S:AtRLK-GFP were transiently transformed into *Arabidopsis* protoplasts. The p16318hGFP vector served as a positive control for construct expression in the transient system. We observed that the GFP control was distributed throughout the cell, while AtAVP1 and AtRLK were expressed on the plasma membrane (**Figure 4**).

### 3.5 AtAVP1 directly interacts with AtRLK *in vivo* and *in vitro*

**Figure 5:**
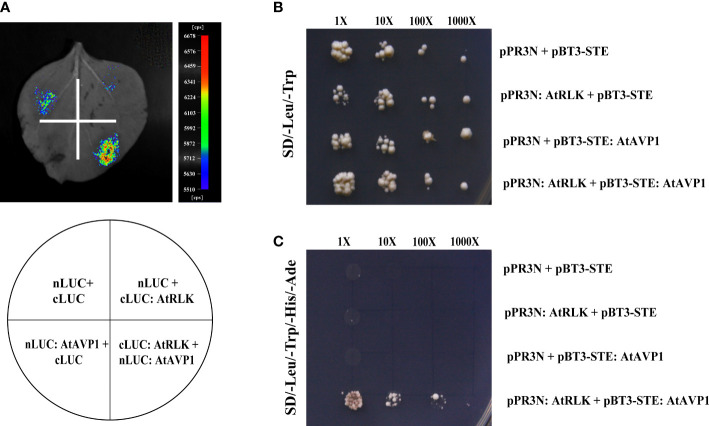
Identification of AtAVP1 interactions with AtRLK **(A)** Identification of an interaction between AtRLK and AtAVP1 using a luciferase complementary imaging (LCI) assay. The nLUC : AtAVP1/cLUC : AtRLK and negative control constructs were transfected into *Agrobacterium tumefaciens* and then injected into four different regions of a tobacco leaf. The leaves were analyzed for luminescence signal by Night SHADE LB 985. **(B, C)** Identification of an interaction between AtRLK and AtAVP1 using a yeast two-hybrid system. **(B)** The pBT3-STE : AtAVP1/pPR3N:AtRLK and negative control constructs were co-transformed into yeast cells and grown on SD/-Leu-Trp double deficiency medium. **(C)** The co-transformed pBT3-STE : AtAVP1/pPR3N:AtRLK and negative control into yeast cells were grown on SD/-Leu-Trp-His-Ade selective medium at 1×, 10×, 100×, and 1000× dilution gradients to test the interaction.

To further verify the interaction between AtAVP1 and AtRLK, we used the luciferase complementary imaging (LCI) assay in addition to the yeast two-hybrid assay, which showed a strong luminescence signal in the nLUC : AtAVP1/cLUC : AtRLK co-injection region. However, no signal was detected in the negative control co-injection region (**Figure 5A**). The yeast two-hybrid results showed that all the fused plasmids grew normally on SD/-Leu-Trp medium (**Figure 5B**), but only pBT3-STE : AtAVP1 and pPR3N:AtRLK could grow normally on the SD/-Leu-Trp-His-Ade selection medium (**Figure 5C**). These results indicate that AtAVP1 physically interacts with AtRLK.

### 3.6 *AtRLK* plays a positive role in the low nitrogen stress response

**Figure 6:**
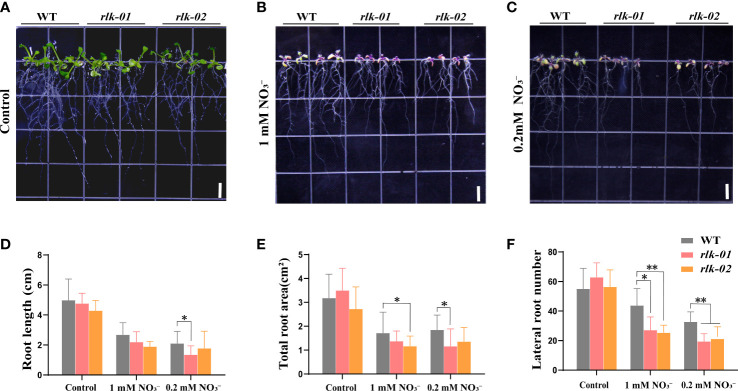
*AtRLK* plays a role in low nitrogen stress tolerance in *Arabidopsis* **(A–C)** Phenotypic analysis of mutant *rlk* and WT plants under control and nitrogen stress conditions. The *rlk*-01 and *rlk*-02 represent the same line plated out twice. **(D)** Root length, **(E)** total root surface, and **(F)** lateral root number in *rlk* mutant lines grown on 6 mM ${\text{NO}}_{3}^{-}$ (control), 1 mM ${\text{NO}}_{3}^{-}$ , and 0.2 mM ${\text{NO}}_{3}^{-}$ media. All data represent mean ± standard deviation (n ≥ 6). Asterisks indicate significant differences compared to WT (* *p<* 0.05, ** *p<* 0.01, Duncan’s multiple range test). Scale bars = 0.5 cm.

To investigate the function of *AtRLK* under low nitrogen stress, the homozygous mutant *rlk* was identified *via* PCR and qRT-PCR according to the insertion location of T-DNA (**Supplementary Figures 1C, E, G**) and grown under the same conditions as *avp1-1*. We found that as the nitrogen concentration decreased, the growth potential of WT and mutant *rlk* gradually weakened (**Figures 6A–C**). Under 1 mM ${\text{NO}}_{3}^{-}$ conditions, the root surface area and the number of lateral roots were significantly lower in *rlk* plants than in WT (*p<* 0.05) (**Figures 6E. F**). Under the 0.2 mM ${\text{NO}}_{3}^{-}$ conditions, the root length, root surface area, and number of lateral roots were significantly reduced compared to those in WT (*p<* 0.05) (**Figures 6D–F**). We observed no significant differences between the *rlk* mutant and WT plants under normal nitrogen conditions (**Figure 6A**).

## 4 Discussion

### 4.1 H^+^-pyrophosphatase positively regulates the response of various plants to low nitrogen stress and other stresses

By analyzing the function of two H^+^-PPases genes with high homology from *Arabidopsis* and *Elymus dahuricus* in response to low nitrogen stress, we showed that H^+^-pyrophosphatase genes can significantly improve plant resistance to low nitrogen stress and have a potentially important application in the field setting. The overexpression of H^+^-PPases genes in *Arabidopsis thaliana* can improve nitrogen use efficiency and protein abundance of H^+^-PPase in *Romaine Lettuce* (Paez-Valencia et al., 2011). Similarly, with the decrease of nitrogen concentration, the expression of *AVP1* increased (**Supplementary Figure 1H**). We used *Arabidopsis* transgenic lines overexpressing *AVP1* and T-DNA insertion mutant *avp1-1* to show that the growth of plants overexpressing *AVP1* in 1 mM ${\text{NO}}_{3}^{-}$ and 0.2 mM ${\text{NO}}_{3}^{-}$ media was higher than that of WT. Meanwhile, mutant *avp1-1* grew to a lesser extent than did WT (**Figures 1B,C, H, I**). We previously constructed transgenic wheat lines overexpressing *EdVP1* (Zhou et al., 2020). To investigate the response of transgenic *EdVP1* wheat to low nitrogen stress, we performed low nitrogen treatment experiments in the greenhouse and field. The greenhouse experiment showed that plant height, root length, aboveground fresh and dry weight, and underground fresh and dry weight were significantly higher than those of WT (*p<* 0.05) (**Figure 2**). The transgenic *EdVP1* lines in field experiments showed increased yield potential, as they had increased grain number per spike under low nitrogen stress (**Figure 3**). These results indicated that *AVP1* improved the low nitrogen tolerance of gramineous crops in the field.

Gramineous crops are the main food source for the global population. With the reduction in nitrogen fertilizer application being pushed world-wide, it is essential to ensure global food security by improving low nitrogen tolerance of gramineous crops. A previous study found that nitrogen starvation induced the expression of *Salicornia europaea VP1 (SeVP1)* and *SeVP2*. *Arabidopsis* and wheat plants expressing *SeVP1* or *SeVP2* were more tolerant to low nitrogen treatment than were WT, which indicated that the overexpression of H^+^-PPase was conducive to the transport of photosynthates into the root system, thus promoting root growth and integrating nitrogen and carbon metabolism (Lv et al., 2015). These findings are consistent with our results, further indicating that H^+^-pyrophosphatase has a promising potential for improving the resistance of *Arabidopsis* and gramineous crops to low nitrogen stress.

Salt stress is a ubiquitous abiotic stress that adversely affects plant growth and development. We found that *AVP1* also modulated salt stress. The relative expression of *AVP1* in *Arabidopsis* increased after treatment with salt (**Supplementary Figure 1K**); additionally, the survival rate of transgenic *EdVP1* wheat treated with 100 mM NaCl was higher than that of WT (**Supplementary Figure 3B, C**). Indeed, the *Arabidopsis* seedlings cultured with 50 and 100 mM NaCl showed enhanced H^+^-PPase activity on the vacuolar membrane (Krebs et al., 2010). The greater salt tolerance observed in transgenic *AVP1* plants may be related to the fact that H^+^-PPase can promote the accumulation of Na^+^ in the vacuole, which inhibits the toxic effects of excess Na^+^ on plant cells (Gaxiola et al., 2001; Shaheen et al., 2008). These results suggest that H^+^-pyrophosphatase genes may play a role in plant responses to various abiotic stresses.

### 4.2 AtAVP1 interacting with AtRLK may associated with an auxin signaling pathway that enhances low nitrogen tolerance in plants

The receptor-like protein kinase (AtRLK) that interacts with AtAVP1 was selected using the yeast two-hybrid system. We found that both *AtAVP1* and *AtRLK* were highly expressed in roots (**Supplementary Figure 1D**), where the proteins are located on the plasma membrane (**Figure 4**). The interaction of AtRLK with AtAVP1 was further determined using yeast two-hybrid and luciferase complementary imaging (LCI) assays (**Figure 5**). To investigate whether *AtRLK* responds to low nitrogen stress, we further studied a T-DNA insertion mutant *rlk* line. Under low nitrogen (0.2 mM ${\text{NO}}_{3}^{-}$ ) conditions, the root length of the *rlk* mutant was shorter and the root surface area and number of lateral roots were lower than those of WT (**Figure 6**). These phenotypes were similar to those of the *avp1-1* mutant, leading to the hypothesis that the two proteins regulate plant responses to low nitrogen stress using the same signaling pathway.

Receptor-like protein kinases (RLK) are important signal transduction network members that play a role in plant response to various abiotic stresses. Moreover, RLK are widely involved in cell signal transduction and plant response to stress (Morris and Walker, 2003; Morillo and Tax, 2006; Lemmon and Schlessinger, 2010). Analysis of the expression profiles of the SsLRR RLK in the root and leaf transcriptomes of the two sugarcane cultivars ROC22 (resistant) and Badila (susceptible) under low nitrogen conditions showed that some genes in the SsLRR RLK gene family were highly expressed, indicating that *RLK* respond to low nitrogen stress (Cheng et al., 2021). RLK can recognize extracellular signals on the cell surface and activate downstream signaling pathways by phosphorylating specific target proteins (Shiu and Bleecker, 2001). We hypothesized that AtRLK functioned upstream of AtAVP1 to regulate the low nitrogen response.

Furthermore, we found that *AVP1* expression increased after exogenously adding different concentrations of IAA (**Supplementary Figure 1J**). It has been reported that plant V-H^+^-PPase activity is involved in root regulation through auxin transport, and *AVP1* overexpression increases auxin transport. The *avp1* deletion mutation severely interferes with root/shoot development and reduces auxin transport (Li et al., 2005), and the *avp1* deletion mutation direct sequencing showed that the T-DNA insertion of this line localizes to the predicted fifth exon of the *AVP1* ORF. Transgenic *AVP1 Arabidopsis* had increased plasma membrane H^+^-ATPase and Pin-formed 1 (PIN1) auxin effector protein activity and auxin content in the germ compared with that in WT (Li et al., 2005). *AVP1* was also found to regulate auxin transport by increasing plasma membrane H^+^-ATPase activity. The root auxin content in *Agrostis stolonifera* expressing *AVP1* was significantly higher than that in WT (Li et al., 2010); based on current literature and our results, we hypothesize that RLK may activate the IAA signaling pathway through the phosphorylation of *AVP1* to improve plant tolerance to low nitrogen conditions. Further studies are needed to elucidate the role of *AVP1* in regulating low nitrogen stress through auxin related pathway. Our findings lay the foundation for further research on improving plant tolerance to low nitrogen stress.

## Data availability statement

The datasets presented in this study can be found in online repositories. The names of the repository/repositories and accession number(s) can be found in the article/**Supplementary Material**.

## Author contributions

HZ completed the experiments, wrote the manuscript, and analyzed the data. HZ, MC, and CX designed the experiments and edited the manuscript. CX helped with the experiments, RL helped with preparing the yeast two-hybrid screening library, YB provided the experimental materials, and WT, KC, ZX, JC, and YM provided the illustration for experiments. DS and HF coordinated the project and edited the manuscript. All authors contributed to the article and approved the submitted version.
