# Supplementary material for: H+-pyrophosphatases enhance low nitrogen stress tolerance in transgenic Arabidopsis and wheat by interacting with a receptor-like protein kinase
Source: Front Plant Sci. 2023 Jan 27;14:1096091. doi: 10.3389/fpls.2023.1096091 (PMC9912985; doi:10.3389/fpls.2023.1096091)
Supplement: Supplementary file 1 [file DataSheet_1.docx]

***Supplementary Materials***

**Supplementary Figures**


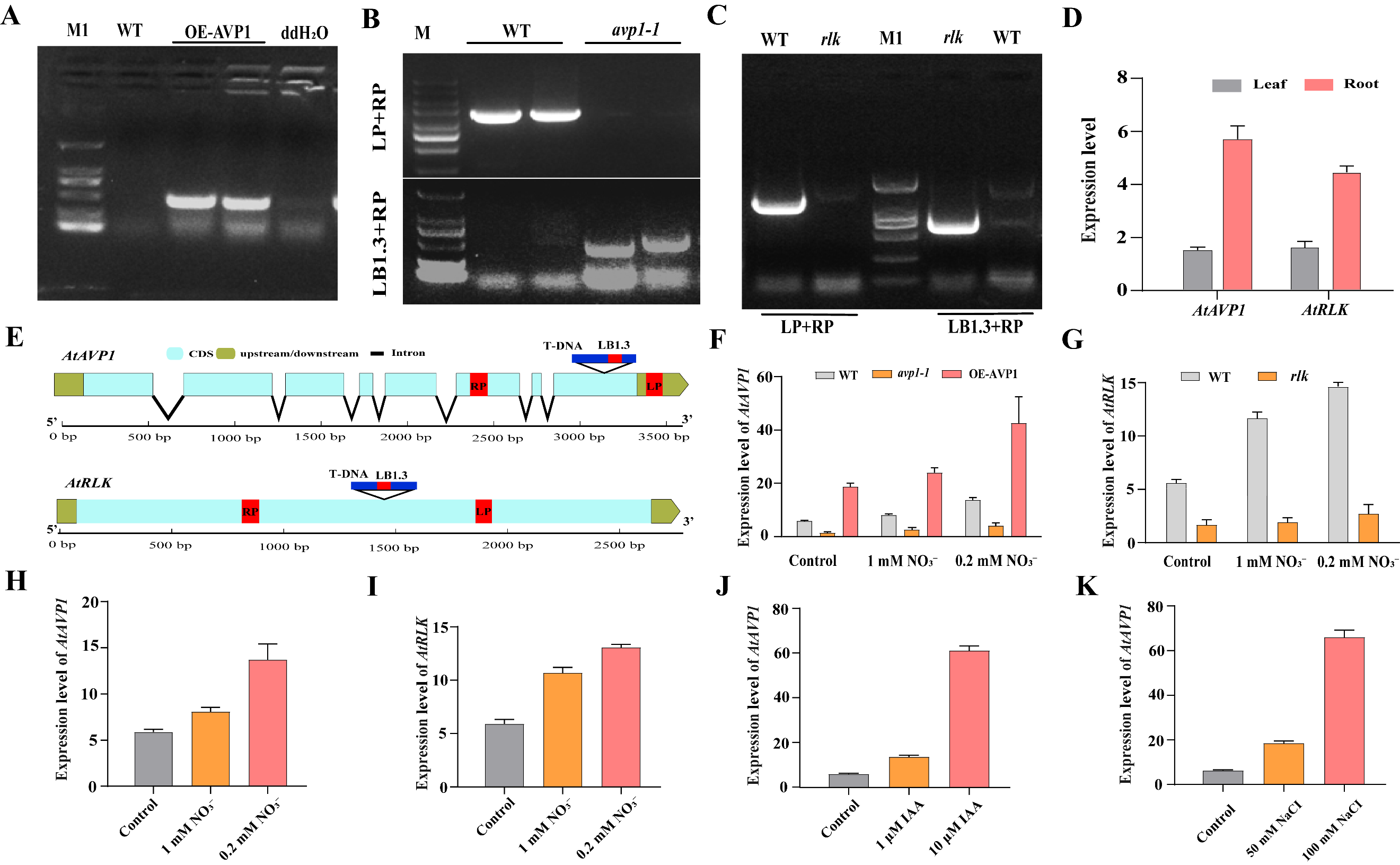


**Supplementary Figure 1.** **PCR identification of transgenic *AtAVP1* (OE-AtAVP1) and mutants *avp1*, *rlk Arabidopsis* lines, and expression analysis of *AtAVP1* and *AtRLK* in tissues and different treatments.** (A) PCR analysis of T_3_ generation transgenic *Arabidopsis*. M1, DNA Marker (BM2000); WT, Wild-type; OE-AVP1, transgenic line; ddH_2_O, negative control. (B) PCR identification of *avp1* mutant*.* LP (*avp1*-LP) and RP (*avp1*-RP) were primers on both sides of the T-DNA insertion site on the plant genome; M, DNA Marker (BM5000); LB1.3 was the primer on the T-DNA segment. (C) PCR identification of *rlk* mutant. LP (*rlk*-LP) and RP (*rlk*-RP) were primers on both sides of the T-DNA insertion site on the plant genome, and LB1.3 was the primer on the T-DNA segment. M, DNA Marker (BM2000) (D) *AtAVP1* (left) and *AtRLK* (right) expression in *Arabidopsis* roots and leaves. (E) Schematic diagram of T-DNA insertion sites of mutants *avp1-1* and *rlk*. (F) *AtAVP1* expression of *avp1-1* mutant line, OE-AVP1 line and WT in roots of *Arabidopsis*, which treated for 5 days under control (6 mM NO_3_^-^), 1 mM NO_3_^-^ and 0.2 mM NO_3_^-^ (low nitrogen) conditions. (G) *AtRLK* expression of *rlk* mutant line and WT in roots of *Arabidopsis*, which treated for 5 days under control (6 mM NO_3_^-^), 1mM NO_3_^-^ and 0.2 mM NO_3_^-^. (H) Expression analysis of *AtAVP1* under control (6 mM NO_3_^-^), 1 mM NO_3_^-^, 0.2 mM NO_3_^-^ conditions in WT roots. (I) Expression analysis of *AtRLK* under control (6 mM NO_3_^-^), 1 mM NO_3_^-^, 0.2 mM NO_3_^-^ conditions in WT roots. (J) Expression analysis of *AtAVP1* under control, 1 μM IAA and 10 μM IAA in WT roots. (K) Expression analysis of *AtAVP1* under control, 50 mM NaCl and 100 mM NaCl in WT roots.


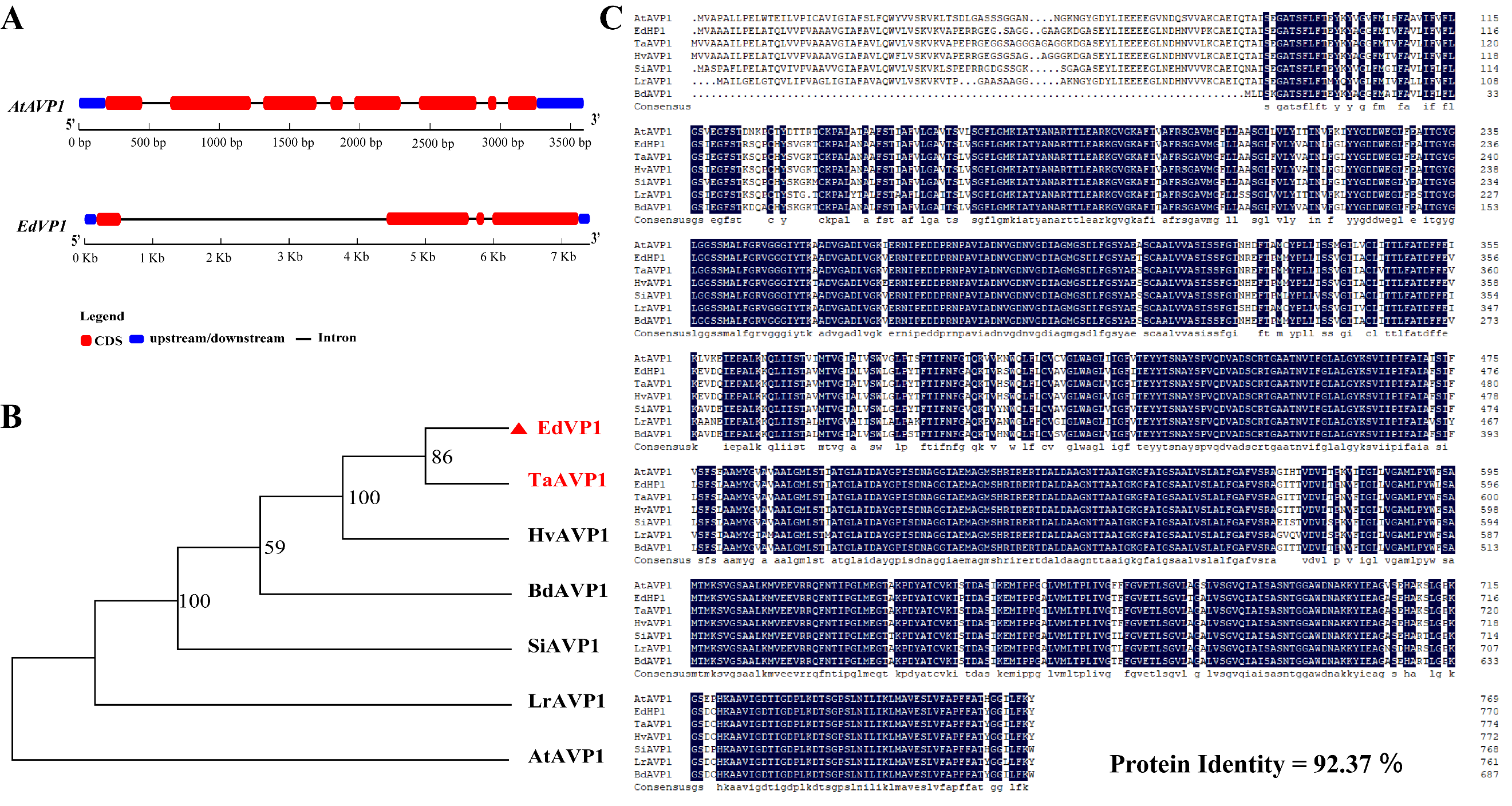


**Supplementary Figure 2. Gene structure, phylogenetic tree and sequence alignment of *AtAVP1* and *EdVP1*.** (A) Gene structure of *AtAVP1* and *EdVP1*. Blue is the untranslated region (UTR). Red is exon. Black is intron. (B) *AVP1* phylogenetic tree. The red triangle is *EdVP1*. Wheat (Ta), *Arabidopsis thaliana* (At), *Hordeum vulgare* (Hv), *Brachypodium distachyon* (Bd), *Lolium rigidum* (Lr). (C) Sequence alignment of proteins with AVP1 homology.


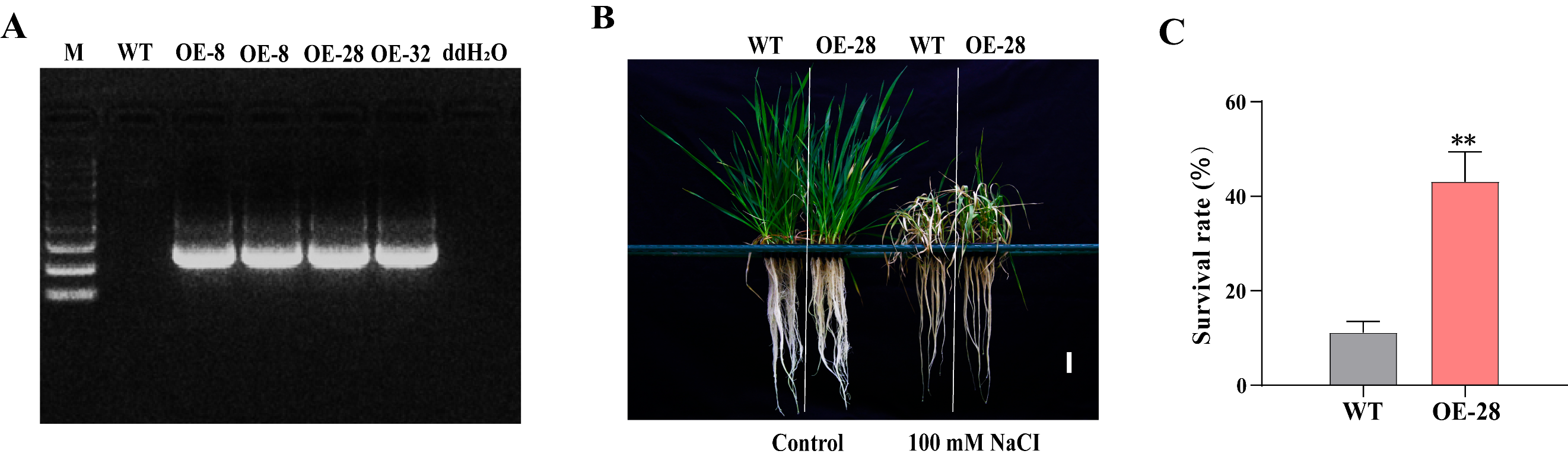


**Supplementary Figure 3. T_3_ generation transgenic *EdVP1* wheat (OE-EdVP1) identified by PCR and the phenotype identification under 100 mM NaCl.** (A) PCR analysis of T_3_ generation transgenic *EdVP1* wheat lines. M, DNA Marker (BM2000); ddH_2_O, negative control; WT, SHI366; OE-8, OE-28, OE-32, transgenic *EdVP1* wheat lines. (B) Phenotypes of SHI366 and transgenic wheat OE-28 under normal and100 mM NaCl condition. Bar= 5 cm. (C) Survival rate of SHI366 and transgenic wheat OE-28 under 100 mM NaCl condition (***p* < 0.01, Duncan’s multiple range test).

**Supplementary Tables**

**Supplementary Table 1. Primers used in this study**

| **Primer** | **Primer sequence (5′-3′)** | **Function** |
| --- | --- | --- |
| AtAVP1-F | CTTATACGGAGGAGAGAAGATGGTGGCGCC | Gene cloning |
| AtAVP1-R | CGGATTGAGTTTAGAAGTACTTGAA |  |
| 16318hGFP-AtAVP1-F | TATCTCTAGAGGATCCATGGTGGCGCCTGCTT | Subcellular localization |
| 16318hGFP-AtAVP1-R | TGCTCACCATGGATCCTCGCGGGCCGG |  |
| 16318hGFP-AtRLK-F | TATCTCTAGAGGATCCATGAAATCAACGTTTCTG | Subcellular localization |
| 16318hGFP-AtRLK-R | TGCTCACCATGGATCCGAAGTGTCCGGCCCGCGA |  |
| pPR3N-AtRLK-F | CGCCTCGGCCTCTCGAGAATTCATGAAATCAACGTTTCTG | Yeast two-hybrid |
| pPR3N-AtRLK-R | CGATAAGCTTGATATCGAATTC TCGCGGGCCGG |  |
| pBT3-STE-AtAVP1-F | TCTGCACTAGGTACCTGCAGATGGTGGCGCCTGCTT | Yeast two-hybrid |
| pBT3-STE-AtAVP1-R | GGGGATCCGTCGACCTGCAGGAAGTACTTGAAAAGGATA |  |
| nLUC-AtAVP1-F | GGACGAGCTCGGTACCATGGTGGCGCCTGCTT | Luciferase Complementary imaging (LCI) |
| nLUC-AtAVP1-R | CGTACGAGATCTGGTCGACGAAGTACTTGAAAAGGATA |  |
| cLUC-AtRLK-F | GTCCCGGGGCGGTACCATGAAATCAACGTTTCTG | Luciferase Complementary imaging (LCI) |
| cLUC-AtRLK-R | TGTTGCTGCAGGTCGACTCGCGGGCCGGACACTTCCT |  |
| qRT-AtAVP1-F | CTGTCATTGCTGATAATGTCGG | Real-time PCR |
| qRT-AtAVP1-R | GATTCCCATTGAACTGATGAGC |  |
| qRT-AtRLK-F | GTTTTGTACGGAAATCGCGATA | Real-time PCR |
| qRT-AtRLK-R | CCATGGTTCATGTACTCGTAGA |  |
| qRT-AtActin-F | TGTTCCCATCAGAACCGTGA | Real-time PCR |
| qRT-AtActin-R | CACCTGTCTTTGGGTCAACAA |  |
| EdVP1-F | GCTATTGCAAGTAGTCTCCGATTAGG | Real-time PCR |
| EdVP1-R | CATCATGATTCTGTCTGCTCCATGCTC |  |
| Edactin-F | CAGTGGAGGTTCTACCATGTTTCC | Real-time PCR |
| Edactin-R | CATGCAAGGCCATGCCATTGTG |  |
| *rlk-*LP | GATTCTCCGGGAAGAATCTTG | Left / Right genomic primer |
| *rlk-*RP | TATGATCTTCTGGTCGCAACC |  |
| *avp1-1-*LP | TTGGAGACACAATTGGAGACC | Left / Right genomic primer |
| *avp1-1-*RP | ATCCACACTCTGTTGCCTTTG |  |
| LB1.3 | ATTTTGCCGATTTCGGAAC | Left border primer of the T-DNA insertion |
| OE-AtAVP1-F | ACTATCCTTCGCAAGACCCT | positive detection |
| OE-AtAVP1-R | GACACCAACATATTTGTACTCCGTG |  |
| OE-EdVP1-F | TCAACGACCACAACGTCGT | positive detection |
| OE-EdVP1-R | GCCGAAAAGAGCCATGGAAGA |  |

**Supplementary Table 2.** ***Arabidopsis* low nitrogen Medium Ingredients**

| **Medium Ingredients (No Nitrogen, Phosphate, or Potassium)** | |
| --- | --- |
| Compound name | Concentration(mg/L) |
| Boric Acid | 6.2 |
| Calcium Chloride, Anhydrous | 332.2 |
| Cobalt Chloride•6H_2_O | 0.025 |
| Cupric Sulfate•5H_2_O | 0.025 |
| Na_2_EDTA•2H_2_O | 37.26 |
| Ferrous Sulfate•7H_2_O | 27.8 |
| Magnesium Sulfate, Anhydrous | 180.7 |
| Manganese Sulfate•H_2_O | 16.9 |
| Molybdic Acid (Sodium Salt)• 2H_2_O | 0.25 |
| Potassium Iodide | 0.83 |
| Zinc Sulfate•7H_2_O | 8.6 |

**Supplementary Table 3. Field data of transgenic *EdVP1* wheat line OE-28, OE-32 and SHI366 plants in 2020 -2021 and 2021-2022**

|  |  | **2020-2021** | | | |
| --- | --- | --- | --- | --- | --- |
|  |  | 1000-grain weight (g) | spike number per m^2^ | Grain number per spike | Grain yield（kg/ha） |
| **Normal** | SHI366 | 45.08±1.96 | 492±8.19 | 31.67±1.53 | 8105.47±299.37 |
|  | OE-28 | 47.12±1.13 | 522.33±12.01 | 32±1 | 8400.81±157.73 |
|  | OE-32 | 46.25±2.90 | 517±16.82 | 32.58±1.83 | 8307±193.36 |
| **LN** | SHI366 | 40.85±2.68 | 441.5±23.40 | 26±1 | 6923.24±306.34 |
|  | OE-28 | 43.12±2.38 | 473±12.77 | 29±1* | 7622.40±263.21* |
|  | OE-32 | 42.58±2.96 | 472.67±6.66 | 28.67±0.58* | 7654.67±287.12* |

|  |  | **2021-2022** | | | |
| --- | --- | --- | --- | --- | --- |
|  |  | 1000-grain weight (g) | spike number per m^2^ | Grain number per spike | Grain yield（kg/ha） |
| **Normal** | SHI366 | 44.16±1.61 | 504.67±6.11 | 33±1 | 8302.97±163.03 |
|  | OE-28 | 47.18±0. 93 | 522±10.58 | 34±1 | 8600.81±182.21 |
|  | OE-32 | 47.12±1.77 | 523.67±13.05 | 34.33±0.58 | 8422.99±229.19 |
| **LN** | SHI366 | 40.50±1.61 | 448.67±14.05 | 27±1 | 7017.24±361.45 |
|  | OE-28 | 42.15±1.85 | 482.33±21.55 | 29±1* | 7660.12±164.68* |
|  | OE-32 | 42.76±1.88 | 479.67±16.50 | 30±1* | 7635.14±170.17* |

**Supplementary Table 4. Some of the results for proteins interacting with AtAVP1 in Y2H**

| **Gene** | **TAIR** | **Function** |
| --- | --- | --- |
| UBP1A | AT1G54080 | mRNA 3'-UTR binding |
| CPuORF35 | AT1G29951 | Upstream open reading frames are small open reading frames found in the 5' UTR of a mature mRNA, and can potentially mediate translational regulation of the largest, or major, ORF. CPuORF35 represents a conserved upstream opening reading frame relative to major ORF AT1G29950.2 |
| RPL18 | AT3G05590 | structural constituent of ribosome, Encodes cytoplasmic ribosomal protein L18 |
| PAS2 | AT5G10480 | protein tyrosine phosphatase activity, enoyl-CoA hydratase activity |
| ENH1 | AT5G17170 | electron carrier activity, metal ion binding |
| CCR2 | AT2G21660 | double-stranded DNA binding, RNA binding, single-stranded DNA binding |
| GER1 | AT1G72610 | germin-like protein (GLP1) |
| TIM | AT2G21170 | triose-phosphate isomerase activity |
| RAB1A | AT5G47200 | GTP binding |
| AGP21 | AT1G55330 | Encodes a putative arabinogalactan-protein (AGP21) |
| AtCDF1 | AT5G23040 | Causes Bax mediated lethality in yeast by generating reactive oxygen species and this effect is suppressed by AtBI-1 |
| AGP21 | AT1G55330 | Encodes a putative arabinogalactan-protein (AGP21) |
| **RLK** | At5G35370 | Receptor-like protein kinase (RLK) is an important member of plant signal transduction network |
| AZK | AT5G11850 | protein serine/threonine/tyrosine kinase activity, kinase activity |
